# Transplant Outcomes in Patients with Idiopathic Membranous Nephropathy

**DOI:** 10.1155/2013/818537

**Published:** 2013-02-27

**Authors:** Claire Kennedy, Carol Traynor, Patrick O'Kelly, Anthony Dorman, Peter J. Conlon

**Affiliations:** ^1^Department of Nephrology, Beaumont Hospital, Dublin 9, Ireland; ^2^Department of Histopathology, Beaumont Hospital, Dublin 9, Ireland

## Abstract

*Background*. The natural history of idiopathic membranous nephropathy and recurrent disease in transplants is variable. We performed a retrospective cohort study of renal transplant recipients with a primary diagnosis of idiopathic membranous nephropathy. We aimed to establish patterns of disease recurrence and to identify factors associated with disease recurrence. *Methods*. We accessed the Irish renal transplant database to identify patients with biopsy-proven idiopathic membranous nephropathy in receipt of a renal transplant between 1982 and 2010. A detailed medical chart review was performed in all cases, and a senior renal histopathologist reviewed all histology specimens. *Results*. The outcomes of 32 patients, in receipt of 36 grafts, are reported. There was a male preponderance (*n* = 29). Significant graft dysfunction, directly attributable to recurrent disease, was evident in 31% of cases at 10 years. There was no significant association between time on dialysis, HLA mismatch, occurrence of rejection, and the development of recurrent membranous disease. One patient was retransplanted twice; all three grafts were lost to aggressive recurrent membranous disease. *Conclusions*. It remains difficult to identify those that will develop recurrent membranous nephropathy. Almost one third of patients in this cohort developed clinically significant recurrent disease at 10 years.

## 1. Introduction

Idiopathic membranous nephropathy is a relatively common cause of nephrotic syndrome in nondiabetic adults. In our centre, 28% of native renal biopsies performed in the setting of nephrotic syndrome yielded a diagnosis of idiopathic membranous nephropathy [1].

The disease occurs most frequently in Caucasian adult males. In females, the diagnosis is more unusual and should prompt consideration of membranous lupus nephritis. An autoimmune basis for idiopathic membranous nephropathy has been established with the recent identification of the M-type phospholipase receptor (PLA2R) as the major antigen [2]. 

Characteristic histological features include diffusely thickened glomerular basement membranes on light microscopy. Immunofluorescence reveals diffuse granular IgG and C3 deposition along the glomerular basement membranes. Discrete subepithelial deposits are visualised on electron microscopy [3]. Histological findings which favour idiopathic membranous nephropathy over secondary disease include IgG4-positive immune complexes and exclusively subepithelial deposits [4]. 

The natural history of idiopathic membranous nephropathy is variable. Spontaneous remission occurs in a significant proportion of patients [5]. The remainder may develop progressive renal failure and may ultimately require renal replacement therapy. Patients are typically risk stratified for disease progression on the basis of renal function and degree of proteinuria [6]. Those at low or intermediate risk of progression are managed conservatively, at least in the short term, with renin-angiotensin-aldosterone blockade, blood pressure control, lipid control and, if specifically indicated, anticoagulation [7]. Various immunosuppressive protocols exist for those at high risk of disease progression. 

Transplantation is the preferred modality of renal replacement therapy in those patients that reach end-stage kidney disease. The disease is known to recur in some transplant recipients and may lead to significant graft dysfunction or failure [8]. Despite the significant burden of membranous nephropathy worldwide, many questions regarding recurrent disease remain. 

We performed a national retrospective cohort study of all renal transplant recipients with a primary diagnosis of idiopathic membranous nephropathy. We aimed to establish patterns of disease recurrence and to identify factors associated with disease recurrence. 

## 2. Methods

Using the Irish national renal transplant database, which contains longitudinal information on all renal transplant recipients in Ireland, we identified a consecutive cohort of patients with idiopathic membranous nephropathy in receipt of a renal transplant between 1982 and 2010. Patients of all ages were included if they had a biopsy-proven diagnosis of membranous nephropathy and lacked clinical or histological features of secondary disease.

Histology was reviewed by a senior renal histopathologist. Many of the light microscopic features of membranous nephropathy, such as basement membrane thickening and tubulo-interstitial change, are not specific for membranous nephropathy. With that in mind, the diagnosis of recurrent membranous nephropathy relied on identification of typical subepithelial immune complex deposits by light or electron microscopy, with characteristic IgG staining on immunofluorescence. Histologic changes were graded (stage I–IV) depending on the location of the immune complex deposits and the extent of glomerular basement membrane thickening.

A detailed medical chart review was performed in all cases. Transplant and patient outcomes were coded and recorded. Acute rejection was defined histologically in accordance with the Banff classification.

All analyses were performed with the use of Stata statistical software, version 10.0. All tests were two-sided, and a *P* value of < 0.05 was considered to be statistically significant. 

## 3. Results

There were 3330 kidney transplants during time period of interest. Patients were excluded if their initial biopsy was unavailable for review or if there were features to suggest secondary membranous glomerulonephritis. Patients were also excluded if they had been lost to follow up during the intervening years. Thirty-two patients remained. The mean duration of followup was 180 months. The majority were male (*n* = 29). The mean age at the time of first transplant was 49.8 years (range 20–68 years). One patient was transplanted in a preemptive fashion; the mean duration of dialysis prior to transplantation was 2.75 years. 

In terms of first transplantation, the majority (*n* = 30) were from deceased donors; three of these were dual kidney transplants from extended criteria donors. Two patients recieved a living related donation. 

All patients had induction immunosuppression in the form of high-dose steroid and an IL-2 receptor blocker. Maintenance immunosppresssion was with low-dose corticosteroid, an antimetablite and a calcineurin inhibitor in combination. Late corticosteroid withdrawal occurred in a minority of patients.

There were four patient deaths, all in the setting of a functioning transplant. The causes of death were cardiovascular disease, gastric malignancy, and lung malignancy in two cases. There were no other reported malignancies among the patient group. 

Eleven transplants were lost during the time period in question. Four cases were due to death with a functioning graft. One case was due to early thrombosis/infarction; another due to severe rejection. Recurrent disease was deemed the major contributing factor to graft failure in five cases (Figure 2). The median time between diagnosis of recurrent disease and graft loss in these cases was 15 months (range 5–49 months).

With regard to posttransplant surgical complications, there was one case of early transplant thrombosis/infarction. There were two cases of anastomotic leak in the early post-operative period. 

Transplant biopsies were performed if clinically indicated to investigate proteinuria, haematuria, or deteriorating graft function. Eighteen patients had a total of 31 transplant biopsies. There were eight cases of biopsy-proven, cell-mediated rejection. The majority of these were Banff Class 1A, managed with steroids and augmented baseline immunosuppression. 

**Figure 1:**
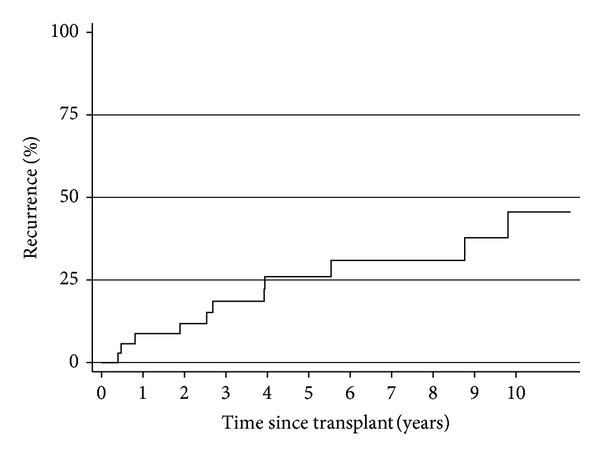
Time to recurrence of membranous nephropathy in first transplants (*n* = 32).

**Figure 2:**
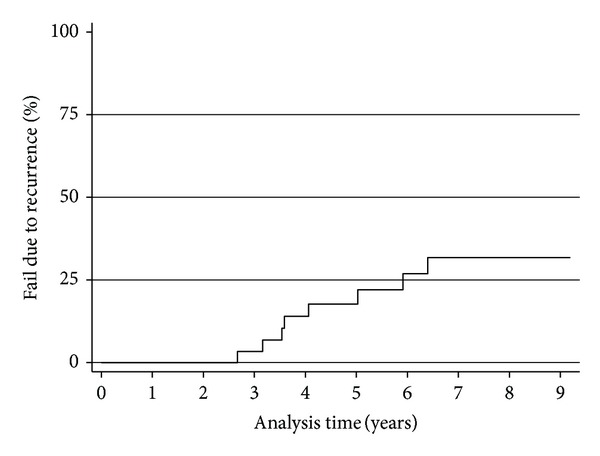
Graft failure due to disease recurrence over time.

Recurrent membranous nephropathy was identified in nine patients. Renal function and proteinuria at the time of biopsy can be seen in Table 2. The median time to recurrence was 46 months with a range of 5–120 months (Figure 1). The majority were treated conservatively (Table 2). One patient, with crescentic recurrence and florid nephrotic syndrome, received rituximab therapy. The nephrotic syndrome, abated and this patient continues to enjoy good graft function three years later.

Three patients underwent renal transplantation for a second time. One case was uncomplicated and continues to enjoy excellent function eight years later. One case was complicated by a urethral stricture requiring intervention. The third case had recurrent membranous disease identified two years after engraftment, which led to graft failure four years posttransplantation. This patient had a third renal transplant from a living related donor. This, again, was lost to recurrent membranous disease at three years. The patient in question continues on home hemodialysis.

We analysed various factors as predictors of disease recurrence. Duration of dialysis, HLA mismatch, PRA % and treatment for acute rejection were not independent predictors of disease recurrence. Age was significantly associated with recurrence, with younger patients more likely to develop recurrence (Table 1). 

## 4. Conclusions

Membranous nephropathy is thought to recur in 30–45% of transplanted patients [9, 10]. Recurrence typically occurs in the second or third year posttransplant [9, 10]. We found a smaller number of recurrences, with a slightly later onset. This was likely due to the fact that biopsies were performed on the basis of clinical indications, and not in a surveillance manner [10]. 

It has proven difficult to identify reliable clinical predictors of recurrent disease. There are conflicting data regarding the theory that living donor transplants are at increased risk of recurrence [11, 12]. Our results indicated that younger age at time of transplant may predict development of clinically significant disease recurrence. This is thought to be due to the fact that younger patients are more likely to have genetic factors underlying disease and also because they live longer therefore have more time to develop recurrence.

As with primary membranous nephropathy, recurrent disease has an unpredictable clinical course. Spontaneous remission has previously been described [13]. The effect of recurrent disease on graft survival varies between studies, but, in general, graft survival appears largely similar to that of other transplanted patients [8]. 

Histopathologic changes can precede clinically evident recurrent disease. A surveillance biopsy study found that recurrent disease may be present with less than one gram of proteinuria [14]. There is evidence that subclinical disease is progressive and therefore may have important clinical implications in time [15]. The role of PLAR antibodies in the identification of recurrent disease has yet to be fully clarified [16]. 

With regard to treatment, there are anecdotal reports of successful resolution of immune deposits and proteinuria with rituximab [9, 10]. However, as the rates of spontaneous remission and disease progression are unclear, the benefits from rituximab administration are ill-defined.

Several issues remain outstanding. Future studies using surveillance biopsies and PLA2R antibodies/antigens may find a predictive model for recurrent disease. Randomised trials comparing rituximab to conservative management are required to further clarify the natural history of the disease and the role of rituximab in its management. 

Our study confirms the clinical importance of this disease—almost one third of transplanted patients with membranous nephropathy have clinically significant disease recurrence at ten years. As long-term renal transplant survival improves, the relative contribution of recurrent disease to graft loss is likely to increase further. Over half of those patients had resulting graft failure within four years of diagnosis. We look forward to future clinical trials which will deepen our insight into this complex disease. 

## Figures and Tables

**Table 1:** The performance of various clinical and immunological parameters as predictors of the development of recurrent membranous nephropathy.

Variable	Hazard ratio	95% confidence interval	
Sex	0.197	0.028	1.37
Age	0.944	0.892	0.998
Time on dialysis	0.889	0.584	1.35
PRA %	1.023	0.988	1.058
HLA mismatch	1.135	0.695	1.85
Acute rejection	0.520	0.111	2.42

**Table 2:** The treatment and outcome of individual patients with recurrent membranous nephropathy.

Gender	Tx	RF-1	P-1	Status Tx1	Rx	RF-2	P-2	Status Tx2	RF-3	P-3	Status Tx3	RF-C	P-C
M	1	96	8	Functioning	Rituximab	—	—	—	—	—	—	104	4.8
M	1	198	9	Functioning	ACEi	—	—	—	—	—	—	120	2
M	2	135	2.4	Failed	ACEi	—	—	Failed*	—	—	—	HD	—
M	3	233	9.35	Failed	ACEi	237	3.75	Failed	200	8.02	Failed	HD	—
M	1	218	4.6	Failed	ACEi	—	—	—	—	—	—	HD	—
F	1	101	4	Functioning	ACEi	—	—	—	—	—	—	116	1.5
M	2	267	4	Failed	Sirolimus	—	—	Died	—	—	—	Died	—
M	1	179	4.5	Failed	ACEi	—	—	—	—	—	—	HD	—
M	1	106	1.5	Functioning	ACEi	—	—	—	—	—	—	120	1

Tx: total number of transplants; RF-1: renal function at time of biopsy of first transplant (umol/L); P-1: proteinuria at time of biopsy of first transplant (g/24 hr); Tx1: first transplant; Rx: treatment instituted for recurrent membranous nephropathy; RF-2: renal function at time of biopsy of second transplant (umol/L); P-2: proteinuria at time of biopsy of second transplant (g/24 hr); Tx2: second transplant; RF-3: renal function at time of biopsy of third transplant (umol/L); P-3: proteinuria at time of biopsy of third transplant (g/24 hr); Tx3: third transplant; RF-C: current renal function (umol/L); P-C: current proteinuria (g/24 hr); ACEi: angiotensin converting enzyme inhibitor; HD: hemodialysis.

*Transplant failed for unrelated reasons.
